# Data Object Exchange (DOEx) as a Method to Facilitate Intraorganizational Collaboration by Managed Data Sharing: Viewpoint

**DOI:** 10.2196/19267

**Published:** 2020-10-27

**Authors:** Ronald G Hauser, Ankur Bhargava, Ronald Talmage, Mihaela Aslan, John Concato

**Affiliations:** 1 Department of Laboratory Medicine Yale University School of Medicine New Haven, CT United States; 2 Center for Medical Informatics Yale University New Haven, CT United States; 3 Information Technology Veterans Affairs Puget Sound Healthcare Seattle, WA United States; 4 Clinical Epidemiology Research Center Veterans Affairs Connecticut Healthcare West Haven, CT United States; 5 Department of Medicine Yale University School of Medicine New Haven, CT United States; 6 Medical Service Veterans Affairs Connecticut Healthcare West Haven, CT United States

**Keywords:** information-seeking behavior, information services, communication media, database, database management system

## Abstract

**Background:**

To help reduce expenses, shorten timelines, and improve the quality of final deliverables, the Veterans Health Administration (VA) and other health care systems promote sharing of expertise among informatics user groups. Traditional barriers to time-efficient sharing of expertise include difficulties in finding potential collaborators and availability of a mechanism to share expertise.

**Objective:**

We aim to describe how the VA shares expertise among its informatics groups by describing a custom-built tool, the Data Object Exchange (DOEx), along with statistics on its usage.

**Methods:**

A centrally managed web application was developed in the VA to share informatics expertise using database objects. Visitors to the site can view a catalog of objects published by other informatics user groups. Requests for subscription and publication made through the site are routed to database administrators, who then actualize the resource requests through modifications of database object permissions.

**Results:**

As of April 2019, the DOEx enabled the publication of 707 database objects to 1202 VA subscribers from 758 workgroups. Overall, over 10,000 requests are made each year regarding permissions on these shared database objects, involving diverse information. Common “flavors” of shared data include disease-specific study populations (eg, patients with asthma), common data definitions (eg, hemoglobin laboratory results), and results of complex analyses (eg, models of anticipated resource utilization). Shared database objects also enable construction of community-built data pipelines.

**Conclusions:**

To increase the efficiency of informatics user groups, a method was developed to facilitate intraorganizational collaboration by managed data sharing. The advantages of this system include (1) reduced duplication of work (thereby reducing expenses and shortening timelines) and (2) higher quality of work based on simplifying the adoption of specialized knowledge among groups.

## Introduction

To help reduce expenses, shorten timelines, and improve the quality of final deliverables, the Veterans Health Administration (VA) and other health care systems seek to promote the sharing of informatics expertise among user groups. This expertise within informatics user groups often develops through individual or small group experience, based on a unique interest or need. Informatics groups with related interests are likely to benefit from each other’s expertise, but only if a mechanism exists to offer, find, and exchange expertise. This ability is called knowledge management, and it is described as a utilized, accessible, and efficient virtual system for knowing who is doing what, how, and with what effects [1]. Some authors believe this ability is rarely available, even in the best health care organizations [1].

Knowledge management is especially difficult in large, as opposed to small, health care organizations. As health care systems grow, they tend to become more “loosely coupled” (impersonal and disaggregated). Loosely coupled health care systems operate with tight functional integration within any unit, but few structures or processes tie the organization’s units together [1]. This scenario has been referred to as a “silo mentality” [2]. Efficiently finding a suitable collaborator also becomes more difficult, because the possible number of collaborators increases with the size of an organization. According to the Metcalfe law, the number of potential collaborators within a health care system has a squared (n^2^) proportional increase [3]. For example, 100 users can form approximately 5000 total collaborations, but 200 users can form nearly 20,000 collaborations. In addition to organizational structure and the combinatorial scale of potential connections between groups, the lack of physical proximity (eg, operation across multiple time zones) and perceived diversity of purpose (eg, financial and patient satisfaction) also represent barriers to collaboration.

Traditional methods to share knowledge have unique considerations when applied to a health care informatics ecosystem. User groups sharing knowledge through the deployment of applications (eg, Docker) will likely generate security concerns. Similar security concerns will also likely exist in sharing source code (eg, GitHub), which could be made into an application. Datasets, as a knowledge-sharing mechanism, may contain protected health information as a necessity. Public data repositories would therefore be reluctant to host data with protected health information (eg, Machine Learning Repository at the University of California Irvine). Transmission of protected health information between groups within a health care system would likely require administrative oversight (such as an approved media of transmission) to mitigate the risk of a data breach. Didactic lectures represent another option to share knowledge, but consumers of shared knowledge may find it inefficient to implement expertise described in a lecture. The VA health care system, while offering many of these existing knowledge-sharing mechanisms, sought additional options.

The VA sought to create an environment where informatics user groups could find and exchange data through a secure channel. To find the desired expertise, users browse a catalog of work, where each element in the catalog represents a database object (eg, database table). They can subscribe to catalog elements of interest, which provides access to the object. Experts publish their work to the catalog or share it privately with other user groups. Permissions are centrally administered, and the exchange of data takes place on a secure database server shared between user groups. Inherent advantages of this system include reduced costs and shortened timelines through a decrease in redundant work. Higher-quality deliverables are a likely result, based on the adoption of specialized, rather than generic, knowledge. The design of the VA’s solution to promote the sharing of informatics expertise (the Data Object Exchange [DOEx]) and statistics on its usage are described.

## Methods

### Overview of the DOEx

**Figure 1:**
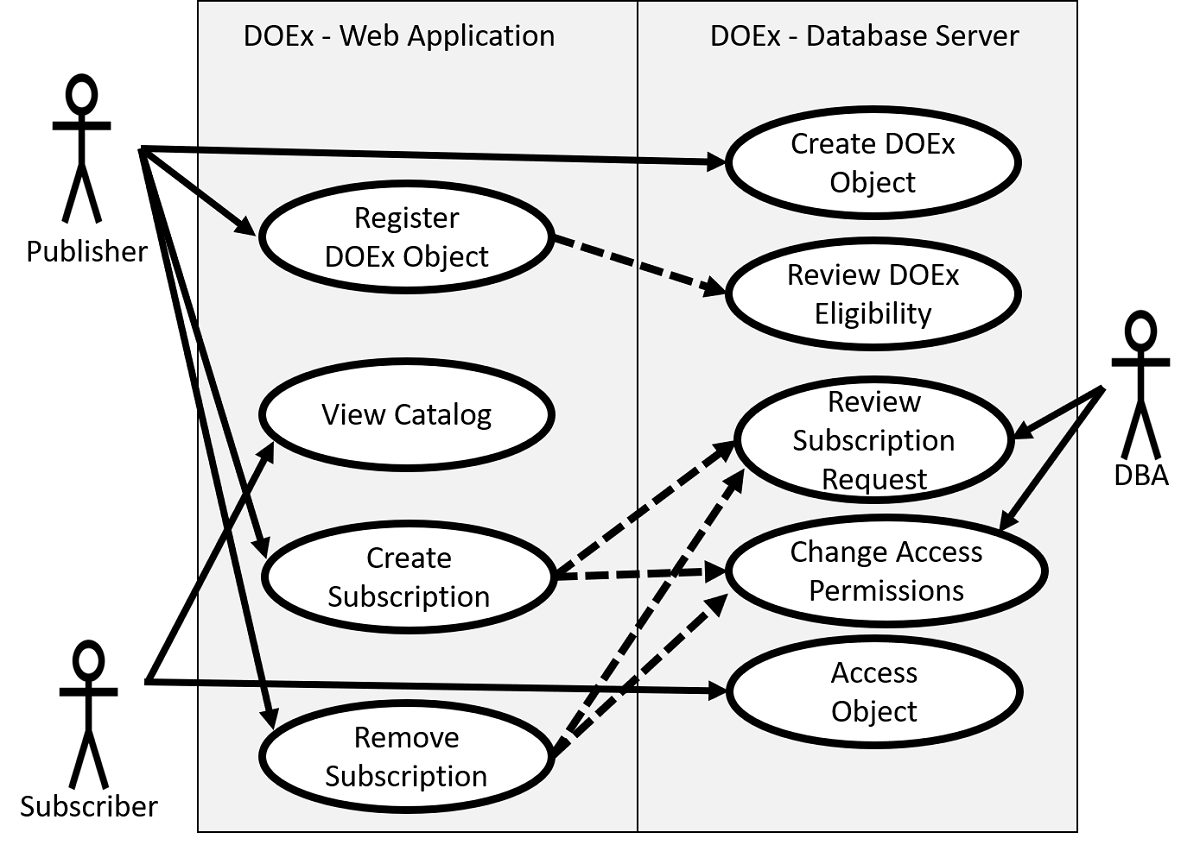
Use case diagram of the Data Object Exchange (DOEx). The DOEx consists of the following two parts: a web application and a database server (gray boxes). Publishers create and register DOEx database objects. Subscribers become aware of shared objects through the DOEx catalog or private communication with the publisher (not shown). Publishers control subscriptions to their DOEx objects through the creation and removal of subscribers. The database administrator (DBA) provides administrative oversight to ensure adherence to the terms of service.

**Figure 2:**
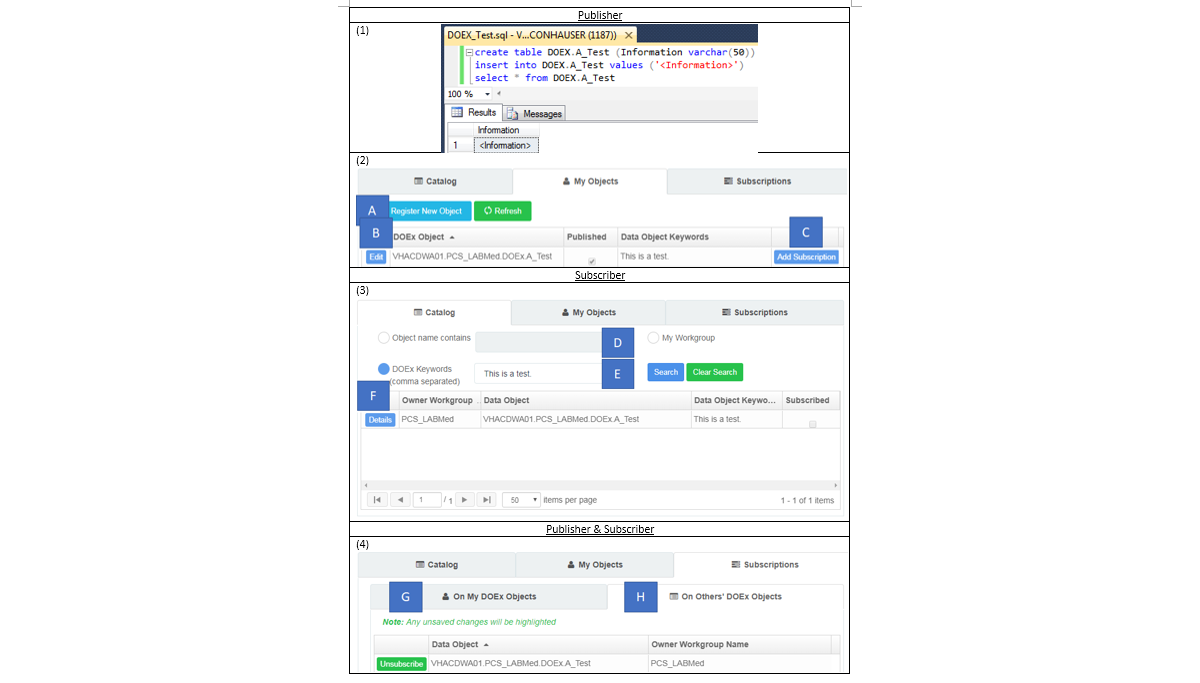
Data Object Exchange (DOEx) web interface. (1) The publisher creates a database object. Example provided in Microsoft SQL Server. (2) The publisher registers an object in the DOEx. (A) “Register New Object” - This button allows the publisher to share the database object via the DOEx. The publisher must provide a description of the object and choose if it will be displayed in the public catalog. (B) “Edit” - Edit the object description and its inclusion in the public catalog. (C) “Add Subscription” - The publisher allows subscribers read-only access to a DOEx object. (3) A subscriber searches the DOEx catalog for objects of interest. (D) Search for public objects by name. (E) Search for public objects by keyword. (F) “Details” - Display the object’s description and publisher’s email. Subscribers email the publisher to request access. The publisher adds the subscriber with 1C. (4) Publishers and subscribers can manage their subscriptions. (G) Publishers can manage subscriptions. (H) Subscribers can manage subscriptions.

To facilitate collaboration, the VA Business Intelligence Service Line (BISL) designed the DOEx. The DOEx operates with a publish-subscribe design pattern (Figure 1) [4]. Similar to popular subscription services (eg, Wall Street Journal and Netflix), a publisher produces content that subscribers consume. In the DOEx, publishers are collections of individuals, operating as workgroups, with a shared goal or interest. Workgroups may publish their work in a catalog, which all other workgroups can browse. Alternatively, workgroups may publish their work through the DOEx without advertising it in the public catalog. Workgroups can subscribe to published objects they find in the public catalog or learn about through private collaborations. In either case, the subscribing workgroup can request a subscription from the publishing workgroup, which provides them read-only access to the requested object. A walkthrough of the workflow used by the publisher and subscriber is shown in Figure 2.

**Figure 3:**
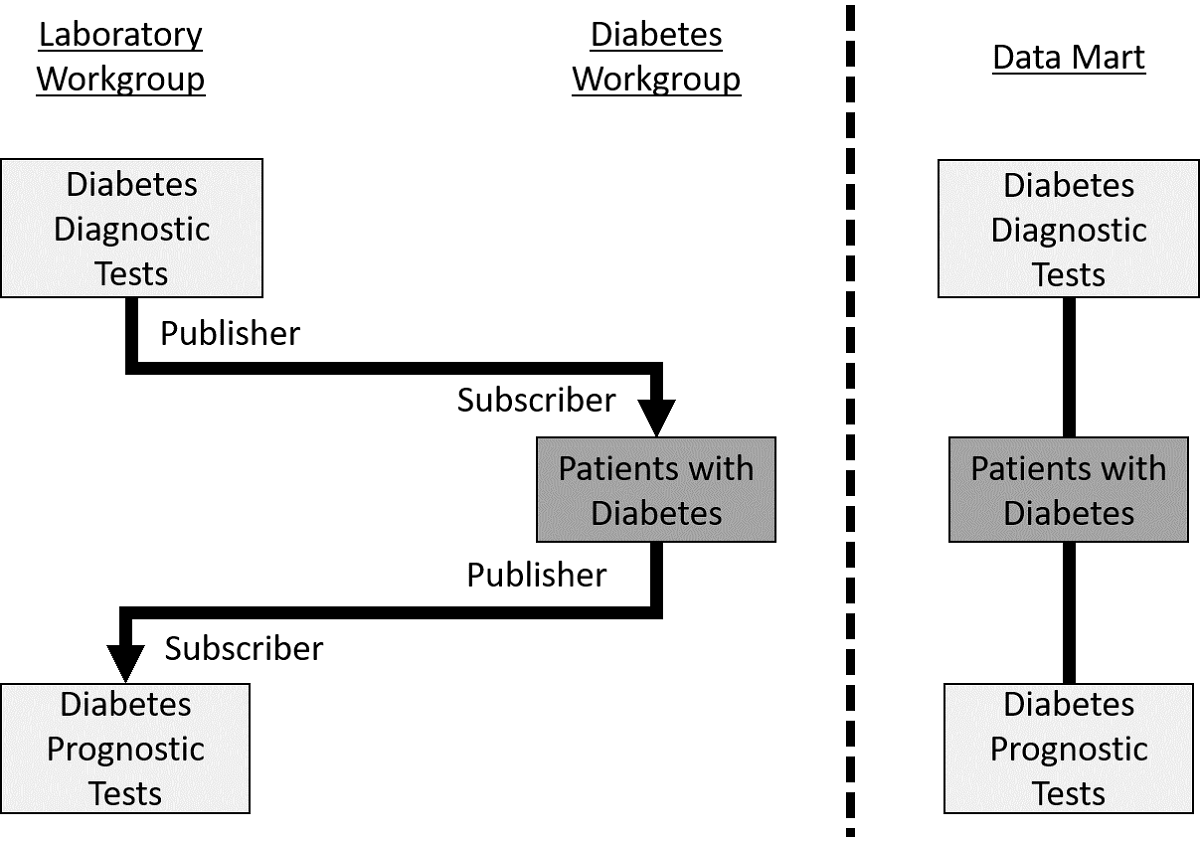
Creating a diabetes data mart with the Data Object Exchange (DOEx) and the dynamic data pipeline design pattern. (Left) The publisher/subscriber relationship between the laboratory and diabetes workgroups. Each rectangle represents a dynamic data design pattern. The arrows denote the data processing sequence. (Right) A subset of the final data mart: a database table of patients with diabetes and their prognostic tests.

The DOEx consists of the following two components: a web application and a database server. The web application allows publishers to manage their subscriptions and control the content they choose to publish. The web application also hosts the catalog of published work and provides metadata about each available subscription (eg, object owner and email address, object description, and object location on the database server). The database server contains a collection of databases, typically one per workgroup. Each workgroup database contains a set of data objects (eg, data tables and views), which may be shared through the DOEx. Tables designed for sharing through the DOEx are placed by the publisher in a database schema named “DOEx.” Additionally, DOEx objects may be designated as either public or private. Public DOEx objects have a record in the DOEx catalog, which advertises them to potential subscribers (Figures 2 and 3). Private DOEx objects, by comparison, do not exist in the DOEx catalog. Private DOEx objects generally form in the context of existing collaborations between publishers and subscribers.

Alongside the publisher and subscriber, a database administrator also participates in the setup and management of object sharing. The web application alerts the database administrator to a publisher-subscriber DOEx request. The database administrator reviews the shared object to ensure compliance with the terms of service of the DOEx. Certain types of data, such as patient names and social security numbers, cannot be shared between workgroups. When these terms are met, the database administrator approves the request.

All modifications to DOEx objects are communicated via email to publishers, subscribers, and database administrators.

### Data Security Considerations

Permissions granted to workgroups vary based on their need to access protected health information and/or personally identifiable information. When subscribers and publishers have different permissions to protected health information/personally identifiable information, they cannot share objects through the DOEx. This prevents the sharing of sensitive data with workgroups that do not have the necessary permissions.

To limit violations of the terms of service, publishers cannot swap one DOEx object for another with the same name. This type of modification inactivates the sharing of the object with its subscribers. Publishers can only swap one DOEx object for another after removing the subscribers and repeating the process of object registration, which requires database administrator review.

In theory, the web application could perform object registration and subscription services, but this approach would require the web application to have elevated permissions on the databases. Given concerns for security, such services are not currently made available.

## Results

### Usage Description

The DOEx went live in May 2017, and as of January 2019, 707 database objects were shared among 758 workgroups. The public catalog contains 217 (30.7%) database objects, and the remaining 490 (69.3%) objects are shared privately; these 707 objects have 1202 subscribers. Of the available DOEx objects, 230 have multiple subscribers, and the 10 most popular DOEx objects have between 10 and 40 subscribers.

### Design Patterns

Experience with the DOEx has led to the emergence of the follow three common DOEx design patterns: static data, dynamic data, and dynamic data pipelines.

The static data design pattern works well for data that does not change or changes very slowly over time. Examples of static data shared through the DOEx include the laboratory test standard known as Logical Observation Identifiers Names and Codes (LOINC), which is updated twice each year. Accordingly, the static data design pattern, the simplest of the three, requires a single DOEx object.

The dynamic data design pattern works well for data that updates frequently. Typical uses for this design pattern include maintenance of study populations, such as an up-to-date list of patients with diabetes in the health care system. This design pattern involves the following two DOEx objects: the “dynamic data table” containing up-to-date data and the “update time table” containing a timestamp of the last update. Subscribers to a dynamic design pattern subscribe to both objects. The subscriber uses the timestamp in the “update time table” as a signal to review the updated “dynamic data table.”

The third design pattern, the dynamic data pipeline, uses two or more dynamic data design patterns in sequence (Figure 3). Users employ this design pattern to maintain an up-to-date data mart [5]. For example, to construct a data mart of patients with diabetes, the first dynamic data design pattern would aggregate all diagnostic tests for diabetes. A second dynamic data design pattern would assemble the patients with diabetes, derived from those diagnostic tests. A third dynamic data design pattern would aggregate the prognostic tests used to monitor the progression of the cohort’s disease. This pipeline executes in a stepwise fashion as follows: the first dynamic data design pattern updates on a schedule, and the remaining dynamic data design patterns update in response. More than one workgroup is likely to contribute to a dynamic data pipeline.

## Discussion

### Summary of the DOEx

This report introduces the DOEx, an application designed to facilitate the sharing of expertise among informatics user groups within the largest integrated health care system in the United States, the VA. The DOEx currently hosts over 1200 securely managed collaborations. These collaborations are entirely voluntary, rather than centrally planned, and presumably exist because they enhance work performance. No other platform to share expertise among informaticists exists at this scale in any other health care system. Although the impact of these collaborations is not formally quantified, by its very nature, the DOEx will reduce redundant work by allowing users to leverage already existing expertise to achieve their objectives. This reduces costs and shortens the time required to produce deliverables.

The DOEx has also likely resulted in higher quality deliverables, as user groups now take advantage of specialized knowledge, which would be prohibitive for them to recreate. An example of this specialized knowledge includes the care assessment needs (CAN) score, a predictive model for death and readmission [6]. Users throughout our health care system can subscribe to the CAN score DOEx object to incorporate this predictive model into their diverse needs. This work includes, for example, the identification of patients at hospital discharge at risk for readmission, which is an operational focus [7]. Alternatively, researchers have explored the relationship between CAN scores and physical function [8].

Implementation of the DOEx by health care systems besides our own could likely be easily achieved. The premise on which the DOEx operates is simple. It securely manages the authorization and therefore the sharing of database objects among workgroups. All major database vendors offer diverse options for denoting authorization, including Microsoft (SQL Server) [9], Oracle (Oracle Database) [10], and IBM (DB2) [11].

Users of the DOEx have reported positive effects on the VA’s informatics ecosystem through allowing DOEx publishers to publicly showcase the content they produce and still maintain control over it. For example, users can register their work in the public catalog, viewable by all users in the health care system, and can unsubscribe users who violate the established collaborative agreement. In this manner, the DOEx promotes a concept deemed psychological ownership (“a bonding such that the organizational member feels a sense of possessiveness toward the target of ownership even though no legal claim exists”) [12]. Researchers have shown this encourages further innovation [13,14]. Additional benefits include extrarole performance (defined as behaviors of employees above their stated job requirements) that promote the smooth functioning of an organization [12,15]. For example, the publisher of a widely subscribed DOEx object may become known throughout an organization as a subject matter expert and thus gain additional motivation to share expertise. Additionally, users have conveyed they will review the public DOEx catalog prior to pursuing their assigned task to mitigate the risk of redundant effort.

In addition to user benefits, the DOEx also facilitates the efficient operation of a health care database. Subscribers can access DOEx objects in place, rather than making a duplicate copy on their workgroup database. This reduces the memory requirements of the database. Similarly, when a subscriber recycles the product of another workgroup, they do not need to create it themselves. This reduces the computational burden on the database. These optimizations benefit all users of the health care system by increasing database performance.

### Alternatives to the DOEx

Consistent with other authors, we found one example of data sharing within a health care system [1]. The Informatics for Integrating Biology and the Bedside (i2b2) Hive operates as a collection of interoperable services. Services are provided by cells that communicate through a web interface [16]. The design of the i2b2 and DOEx appear to have different use cases. The focus of i2b2 is research since it operates as a National Institutes of Health–funded National Center for Biomedical Computing (NCBC). In contrast, the DOEx was conceived to support operational work, rather than research, within the VA.

### Limitations

The DOEx, given all its stated advantages, also has limitations. The publisher-subscriber workgroups must form and maintain an element of trust. This trust can be fostered early in the collaboration, ideally prior to a subscription, by defining the relationship such as a “terms of use” or software license (eg, Apache and GNU General Public License [GPL]).

Subscribers must also trust the content of publishers. The DOEx does not require the exchange of the methods used to create database objects; therefore, some user groups may find it difficult to incorporate work from another group without additional details on the methods.

The DOEx is currently used only by the operations community, including individuals tasked with supporting the day-to-day business operations of the VA. It is not available within the VA research community at this time, given that peer-to-peer sharing of expertise is prohibited on the research database server. Deployment of the DOEx within the research community in the future may facilitate and expand collaboration.

The DOEx also provides read-only (unidirectional) access by subscribers from publishers. Modification of the DOEx to allow read and write (bidirectional) access to DOEx objects would allow subscribers to request individualized output from a publisher. Web services utilize this type of bidirectional communication, such as the model for collaboration found in the i2b2.

At present, the majority of DOEx objects (490/707, 69.3%) are shared privately. The DOEx catalog may not currently contain the breadth of available expertise, and consequently, experts may hesitate to offer their expertise via the DOEx. Improving engagement and awareness of the DOEx (eg, email communications and presentations) will likely improve the number, scope, and quality of offerings in the catalog.

### Conclusion

Sharing of expertise within a health care system’s informatics community includes the need to develop a workflow allowing workgroups to find, offer, and exchange expertise in a secure manner. To address this need, the DOEx promotes shared informatics expertise across workgroups within a health care system, and it reduces costs and shorten timelines through a decrease in redundant work. The DOEx also produces higher-quality deliverables, based on the adoption of specialized knowledge.

## Abbreviations

CAN: care assessment needs
DOEx: Data Object Exchange
VA: Veterans Health Administration
